# Extraribosomal Functions of Bacterial Ribosomal Proteins—An Update, 2023

**DOI:** 10.3390/ijms25052957

**Published:** 2024-03-03

**Authors:** Leonid V. Aseev, Ludmila S. Koledinskaya, Irina V. Boni

**Affiliations:** Shemyakin-Ovchinnikov Institute of Bioorganic Chemistry RAS, 117997 Moscow, Russia; leroymail@gmail.com (L.V.A.); tchufistova@yandex.ru (L.S.K.)

**Keywords:** bacterial ribosomal proteins, extraribosomal functions, RNA–protein interactions, protein–protein interactions, regulation of gene expression

## Abstract

Ribosomal proteins (r-proteins) are abundant, highly conserved, and multifaceted cellular proteins in all domains of life. Most r-proteins have RNA-binding properties and can form protein–protein contacts. Bacterial r-proteins govern the co-transcriptional rRNA folding during ribosome assembly and participate in the formation of the ribosome functional sites, such as the mRNA-binding site, tRNA-binding sites, the peptidyl transferase center, and the protein exit tunnel. In addition to their primary role in a cell as integral components of the protein synthesis machinery, many r-proteins can function beyond the ribosome (the phenomenon known as moonlighting), acting either as individual regulatory proteins or in complexes with various cellular components. The extraribosomal activities of r-proteins have been studied over the decades. In the past decade, our understanding of r-protein functions has advanced significantly due to intensive studies on ribosomes and gene expression mechanisms not only in model bacteria like *Escherichia coli* or *Bacillus subtilis* but also in little-explored bacterial species from various phyla. The aim of this review is to update information on the multiple functions of r-proteins in bacteria.

## 1. Introduction

### 1.1. Ribosome Structure and Functions

Ribosomes are huge ribonucleoprotein complexes that synthesize proteins in all living cells, which is fundamental for life. Ribosomes consist of two subunits: a small 30S subunit and a large 50S subunit (in bacteria); their association results in the formation of a 70S ribosome that is active in translation. A 30S subunit comprises a sole RNA molecule, 16S rRNA, and about 20 different ribosomal proteins (r-proteins), e.g., 21 r-proteins in *E. coli*. A large bacterial 50S subunit contains two molecules of rRNA, 23S rRNA, and 5S rRNA, and more than 30 (33 in *E. coli*) r-proteins

The ribosomal subunits carry out different functions in protein synthesis. The 30S subunit is responsible for the recognition and binding of mRNA during translation initiation, decoding information borne by mRNA, and maintaining the reading frame during protein synthesis; it provides a space for interaction of the mRNA codon with the tRNA anticodon in a decoding center. The large 50S subunit does not form contacts with mRNA and is directly involved in the catalysis of the peptidyl transfer reaction in the peptidyl transferase center (PTC), translocation along mRNA, and it ensures the exit of the growing polypeptide chain through the exit channel. The 50S subunit accommodates the universal CCA tails of tRNAs carrying an amino acid or the growing protein chain and provides the binding sites for protein factors assisting in the initiation, elongation, and termination steps. During a translation cycle, tRNAs occupy consecutively the A, P, and E sites located on both subunits.

Recent advances in cryo-electron microscopy (cryo-EM) and high-resolution X-ray analyses have provided numerous detailed structures of ribosomes from diverse sources and in different conformational states, resolved to near-atomic resolutions. These structures allow us to understand how r-proteins and rRNA regions are arranged in the most important functional centers, how ligands (mRNA, tRNAs, translational factors, and antibiotics) are positioned, and how they interact with the ribosomal components [1,2,3,4,5,6,7,8].

We still know little about the origin of ribosomes and their evolution; this issue is a matter of discussion [9,10,11,12,13,14]. It is widely accepted that the molecular mechanisms of peptide chain synthesis emerged in the RNA world and that the most evolutionarily ancient part of the ribosome is the peptidyl transferase center (PTC). The PTC is almost exclusively composed of RNA, even in the extant ribosome, thus representing a relic of the early steps of the evolution of the translation machinery [9,10,11]. A separate evolution of the peptidyl transferase and decoding functions has been suggested [12]. However, it is not easy to imagine how translation could have evolved from a primitive RNA world because an extant ribosome absolutely requires r-proteins for functioning.

The question about the evolutionary step during which r-proteins are associated with rRNA remains unresolved. Given the surprising structural diversity of r-proteins, it is broadly accepted that the most ancient among them have co-evolved with rRNA to maintain their active conformation, while “younger” r-proteins could be recruited from other processes to improve the quality and fidelity of protein synthesis [9,10,13,14]. R-proteins are among the most abundant proteins in bacterial cells [15]. They account for about one-third to one-half of the molecular mass of the modern bacterial ribosome and, as a rule, are essential for translation. Although deleting the genes for some proteins does not lead to lethality (such proteins are referred to as nonessential), this may cause various growth defects in certain conditions [16,17,18,19]. R-proteins are highly conserved molecules, and for many of them, conservation can be traced from bacteria to humans. Thus, 15 of the 30S r-proteins and 19 of the 50S subunit r-proteins are universally conserved, and according to a new nomenclature [20], they are designated as uS2, uS4, uL1, etc. Other r-proteins are bacteria-specific and are designated as bS1, bS6, bS21, bL9, etc.

As structural components of ribosomes, most r-proteins are capable of RNA binding, and some of them can bind DNA, suggesting their cooptation at later evolutionary steps. Within the ribosome, r-proteins interact not only with rRNA but also with other r-proteins, displaying their capacity for protein–protein interactions. Given their high abundance in cells, r-proteins have the potential to form functional complexes with RNA or protein molecules outside the ribosome, showing extraribosomal or moonlighting activities, which will be discussed in this review.

### 1.2. Arrangement of the r-Protein Genes on Bacterial Chromosomes

Genes encoding r-proteins are organized in operons (21 in *E. coli*) that may include one (e.g., *rpsT*, *rplY*, and *rpmE*), two (e.g., *rplU-rpmA*, *rplM-rpsI*, and *rpmB-rpmG*), or several genes (up to 11 genes, as in the *spc* or *S10* operons). Rather often, the r-protein operons comprise genes encoding non-ribosomal proteins such as translation factors (*tsf*, *fus*, *tufA*), components of the replication complex (*dnaG* and *priB*), or subunits of RNA polymerase (*rpoA*, *rpoB*, *rpoC*, and *rpoD*). This suggests the close interrelationship of the main processes involved in the realization of genetic information, as well as the necessity of their coordination in bacterial cells. In addition, some operons include genes encoding enzymes participating in the modification and processing of tRNA (*trmD* and *rnpA*), the maturation of rRNA (*rimM*), and protein export (*secY*). The biological sense of including these genes in the r-protein operons is not fully clear. In some cases, the non-ribosomal genes are regulated independently of the r-protein genes [21,22].

**Figure 1:**
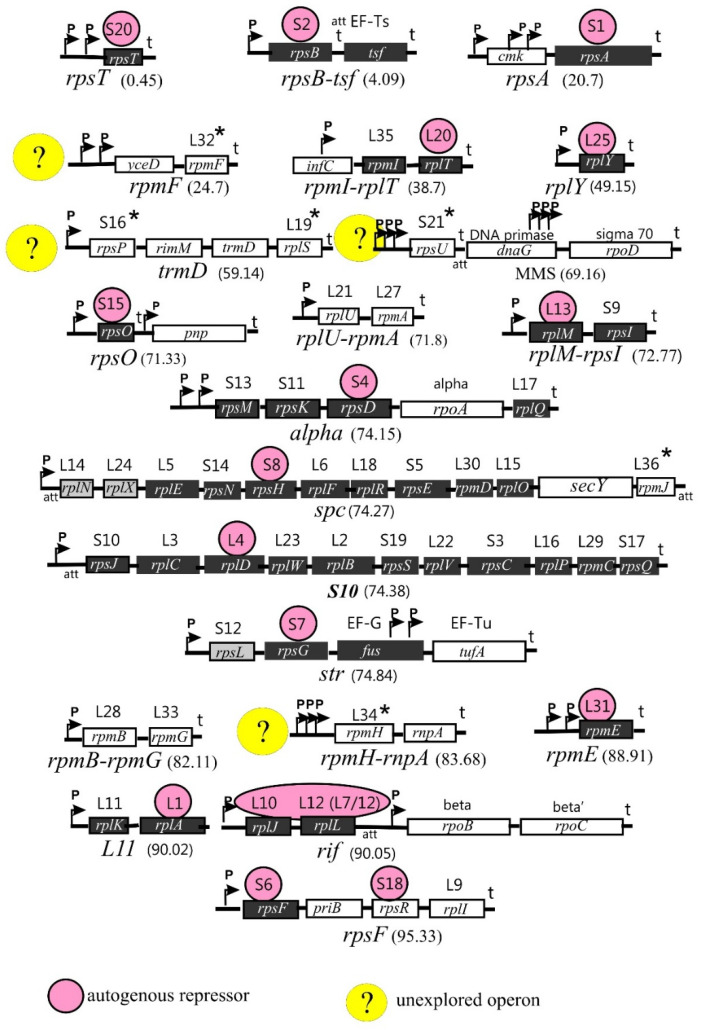
Composition of r-protein operons and their distribution on the *E. coli* chromosome (updated from [22]). The name and position on the chromosome map (in centisomes) for each operon are indicated; r-proteins regulating their own expression are encircled (pink); r-proteins with unknown regulation are marked with asterisks; and yet-unexplored operons with yellow circles. Black background indicates r-protein genes regulated by r-protein-repressors; gray—by the mechanism of retroregulation; white—non-regulated or unstudied genes. P—promoter, t—terminator, att—attenuator.

The structure and distribution of r-protein operons on the *E. coli* chromosome are shown in Figure 1. Although the operon structure is mainly conserved across the bacterial kingdom, it may be rather divergent depending on the taxonomic group. Moreover, one or more r-protein genes may be missing in some prokaryotic genomes [23]. These aspects, if necessary, will be considered in the subsections below.

Ribosome biogenesis requires the coordinated synthesis of all ribosomal components in stoichiometric amounts and, hence, must be tightly controlled both at the transcriptional and translational levels. One of the control mechanisms that play a key role in maintaining the stoichiometry of rRNA and r-proteins is the autogenous regulation of r-protein synthesis [21,22,24,25]. The ability to regulate the expression of its own mRNA by acting as an autogenous repressor is the most characteristic (but not the only one) moonlighting activity of r-proteins, and most r-protein operons include a gene encoding the r-protein-repressor (Figure 1). Often, the r-protein-repressor uses the same RNA-binding site to bind rRNA during the ribosome assembly and its own mRNA to control its expression. Moreover, in several cases, there exists a visible similarity in the structure of both RNA targets, which is referred to as a principle of “molecular mimicry” [26]. Most but not all r-protein operons are feedback-regulated. For instance, the expression levels of the *rplU-rpmA* and *rpmB*–*rpmG* operons do not respond to increased synthesis of their products [27]; in Figure 1, these operons have a white background but are not marked with a yellow circle.

The specific functions of individual r-proteins within the ribosome are often unclear, although recent studies have greatly advanced our knowledge of the activity of r-proteins in translation. In this review, we discuss the moonlighting activities of r-proteins along with their functions within the ribosome in case they have been revealed. The review is compiled in the form of a catalog, where each moonlighting r-protein is discussed in a separate subsection. Each r-protein is named according to [20], where “u” designates a universally conserved protein and “b” is bacteria-specific.

## 2. Moonlighting r-Proteins of the 30S Ribosomal Subunit

### 2.1. Multiple Activities of bS1

#### 2.1.1. Structure and Unique Features of bS1

bS1 is a real champion among all other r-proteins in terms of the number of functions it performs in the cell, uninfected or infected with various bacteriophages (reviewed in [22,28,29]). bS1 is the largest r-protein (557 amino acid residues in *E. coli*); it is an integral and essential component of translation machinery in all members of Proteobacteria, Cyanobacteria, Actinobacteria, and many other bacterial phyla, but it is absent from the ribosomes of Gram-positive bacteria with a low GC content (e.g., Bacillus) [30]. Although the *rpsA* gene encoding bS1 is present in the same context as in *E. coli*, it is not essential, and the function of its product remains unknown. In Gram-negative bacteria, bS1 is essential [31] and consists of six homologous repeats (72–75 amino acid residues each) known as S1 motifs or S1 domains [28,30]; the few known viable bS1 mutants lacking one or two C-terminal domains cause significant growth defects [28,29,32,33].

The S1 domain adopts an OB-fold that is highly specific for binding single-stranded nucleic acids. The OB-fold is an ancient, evolutionary conserved module found in many RNA-associated proteins from bacteria to humans [34]. The two N-terminal S1 domains (D1–D2) lost their RNA-binding functions during evolution and acquired an ability to form protein–protein interactions, while the C-terminal domains (D3-D6) provide the RNA-binding capacity of bS1. bS1 binds to the 30S subunit at the final step of the assembly via domains D1–D2 that form contacts with r-proteins, especially with uS2 [35,36], and its extended, flexible C-terminal part is exposed in solution to provide mRNA binding [28,29]. Interestingly, in hibernating 100S ribosome particles formed by 70S ribosome dimerization under stress conditions, bS1 has a compact conformation with domains D4-D6 folded back to the 30S surface. This inactive conformation is stabilized by the ribosome modulation factor (RMF) that binds to the domain D4 of bS1 to sequester the anti-Shine-Dalgarno (anti-SD) sequence at the 3′ end of 16S rRNA, thereby inhibiting translation initiation [37].

#### 2.1.2. Functions of bS1 in Translation, Translational Control, Transcription, and RNA Decay

The vital function of bS1 as a component of the 30S subunit is the recognition and binding of various mRNAs at the first step of translation initiation [31]. bS1 does not have strict sequence specificity and binds most leadered mRNAs (including heterologous mRNAs), regardless of the presence of SD sequences or secondary structures in their 5′UTRs [31,33,38,39,40,41,42], being dispensable only for leaderless mRNAs [43]. Targets for bS1 are situated within mRNA leaders 5′ to the SD element (if it is present) [29]. Although lacking strict sequence preferences, bS1 has a higher affinity for U- or AU-rich sites, and such S1 targets may serve as translational enhancers [38,40,41]. Another type of high affinity bS1 target is the pseudoknot structure [44,45]. 

Translational enhancers bound by bS1 are essential elements that ensure efficient translation of mRNA and provide a pathway for its regulation. Thus, the U-rich enhancer of the *manY* mRNA can be targeted by a small RNA SgrS, which interferes with efficient translation, suggesting that the sRNA-mediated enhancer silencing could be a common mode of gene regulation [46]. Recent data show that the mRNA-binding specificity of bS1 can be changed by the acetylation reaction in response to nutrient starvation [47]. Under stress conditions, acetylation of the lysin residues K411 and K454 in the domain D5 allows bS1 to selectively recruit a subset of stress-responsive mRNAs, simultaneously lowering its affinity to mRNAs responsible for rapid growth, thus highlighting the role of bS1 in the ribosome-mediated cellular response to stress [47].

Despite the lack of strict sequence preferences, bS1 is a highly specific autogenous repressor that distinguishes its own mRNA from all the others [32,48,49]. bS1 synthesis is strictly feedback-regulated at the translation level due to specific sequence/structure features of the *rpsA* mRNA translation initiation region (TIR), which are highly conserved in several families of γ-proteobacteria [48,49]. In these species, the *rpsA* TIR has a specific fold and lacks a canonical SD element, so the formation of the 30S initiator complex strongly depends on the S1-mRNA interaction. The mechanism of the autogenous regulation is based on competition for the TIR between free bS1 and bS1 bound to a 30S subunit. The transformation of a weak SD to a canonical SD sequence completely abolishes autogenous repression by allowing a 30S subunit to win [48]. The preferential binding of bS1 to its own mRNA is most likely explained by the cooperative interaction of several bS1 molecules with the AU-rich single-stranded regions in the 5′UTR of the *rpsA* mRNA [48,49]. The domain D6 of bS1 appears to be indispensable for its activity as an autogenous repressor [32].

In addition to its role as a highly specific autogenous repressor, bS1 has other moonlighting activities outside the ribosome. bS1 can associate with RNAP and stimulate transcriptional activity by promoting transcription cycling and processivity, with the domains D5 and D6 being involved [50,51]. It has been reported that bS1 may act at the interface of translation and mRNA decay, and its overexpression can protect a set of mRNAs from degradation in *E. coli* [52,53]. At the same time, in *Caulobacter crescentus*, bS1 has been found as an accessory protein that participates in the RNA degradosome assembly at low temperatures and promotes RNA destabilization [54].

#### 2.1.3. bS1 and Trans-Translation

RNA-binding features of bS1 provide its binding with almost all RNAs in vitro, raising the question of whether the observed interaction is biologically relevant. An example of such a problem is the involvement of bS1 in *trans*-translation, which is a remarkable pathway controlling the quality of mRNAs and synthesized proteins in bacteria [55,56,57]. A key player in *trans*-translation is a transfer-messenger RNA (tmRNA, SsrA) that combines the properties of mRNA and tRNA in that it is charged with alanine and contains a short ORF encoding a tag-peptide. Four proteins have been suggested to participate in tmRNA functioning as follows: tmRNA-specific SmpB, alanyl-tRNA synthetase, EF-Tu, and bS1, with the role of bS1 remaining questionable up to now; pro et contra arguments have been reported [58,59,60], with weighty arguments against the possible involvement of bS1 in the tmRNA-mediated quality control in *E. coli* [59,60].

In *E. coli, trans*-translation is not the sole way to rescue ribosomes from nonstop mRNAs, while in some bacteria (e.g., mycobacteria), this pathway is essential for viability [56,57]. It has been proposed that bS1 plays a critical role in *trans*-translation in *Mycobacterium tuberculosis* (*Mtb*) and that this essential pathway might serve as a target for pyrazinamide (PZA), a first-line drug in tuberculosis treatment [61]. Within a living cell, PZA transforms into a biologically active derivative, pyrazinoic acid (POA), that may target *Mtb* S1 at the beginning of a C-terminal extension specific only for Actinobacteria. The binding of POA to *Mtb* bS1 inhibits *trans*-translation, thereby affecting pathogen viability [61]. These findings have attracted much attention and promoted studies of the *rpsA* polymorphism in PZA-resistant strains [62]. However, recent data have provided evidence that *trans*-translation in *Mtb* is not inhibited by PZA or its active metabolite POA, in vitro or in vivo, and moreover, the action of POA turned out to be entirely independent of *Mtb* bS1 [63].

#### 2.1.4. Functions of bS1 during Infections with Bacteriophages

The ability of bS1 to bind to both proteins and nucleic acids underlies its multiple functions during phage infections. Different bacteriophages recruit bS1 for diverse phage-specific processes. bS1 is one of the four subunits of the Qβ phage RNA replicase, as well as replicases of other RNA phages [29,64,65]. Historically, it was the first discovered moonlighting activity of a ribosomal protein [64]. The role of bS1 in the replication of Qβ RNA has been thoroughly studied by various biochemical and structural approaches [66,67,68,69]. bS1 is strictly required for the initiation of replication of the Qβ RNA-positive strand but dispensable for replication of a negative strand. The two N-terminal domains, D1-D2, anchor bS1 onto the phage-specific β-subunit, and the third domain, D3, is mobile and protrudes beyond the surface of the β-subunit to interact with phage RNA [66,67]. bS1 does not appreciably influence the rate of elongation during replication of Qβ RNA but is necessary for the termination of RNA synthesis. The N-terminal domains D1-D3 appear sufficient for an efficient release of a single-stranded RNA product from the template RNA [68,69].

bS1 has been found to form a strong complex with the λ phage β protein that is a component of the Red pathway of the phage recombination system [70]. Although this could suggest the role of bS1 in *red* recombination events, this effect was not further studied. The most intriguing findings concern multiple activities of bS1 during T4 bacteriophage infection. bS1 has been found to stimulate (by a factor up to 100) activity of the T4 endoribonuclease RegB that inactivates some early phage mRNAs by cleaving in the middle of the SD sequence GGAG [71,72,73,74,75]. The minimal domain combination required for stimulation of RegB is D4–D5, whereas all C-terminal domains (D3–D4–D5–D6) stimulate RegB to the same extent as the full-length protein. Given that direct interactions between RegB and bS1 have not been detected, and RegB has only a low affinity for its RNA substrates, it has been suggested that bS1 stabilizes the mRNA–RegB complex during a primary step of mRNA binding [75]. 

**Figure 2:**
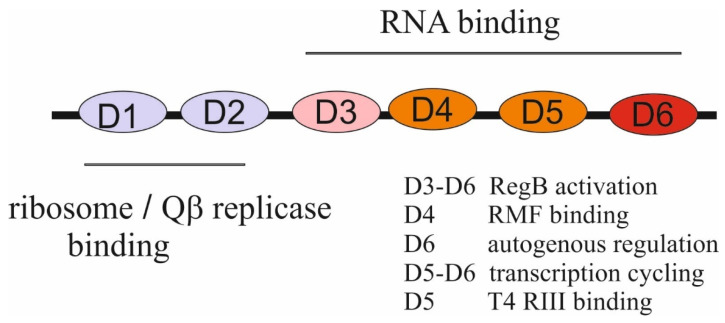
Modular structure of *E. coli* bS1 and involvement of its domains in diverse interactions and processes in a cell. Colors of the domains reflect differences in their functions.

While the RegB activity is activated by bS1, this activation may be abolished by the T4-encoded RIII protein known as a cytoplasmic antiholin [76]. Direct protein–protein interactions between bS1 and RIII have been characterized, suggesting that RIII may interfere with the biological activities of bS1 in infected cells. RIII appears to be the first effector protein of the T4 phage, which targets bS1 at its RNA-binding domains, mainly at the domain D5 [76]. One more remarkable finding concerning the T4-mediated processes in *E. coli* has been recently reported. When T4 infects *E. coli*, it modifies the translational apparatus of the host by using the adenosine diphosphate (ADP)-ribosyltransferase ModB that can attach entire NAD-capped RNA chains to acceptor proteins in an ‘RNAylation’ reaction [77]. ModB specifically RNAylates bS1 at arginine residues R139 and R142 in the domain D2 by using selected NAD-capped *E. coli* and T4 RNAs. As the authors suggest, the ModB-mediated RNAylation of r-proteins may be one of the molecular mechanisms used by the T4 phage to target the translational machinery of its host [77]. Interactions of different domains of bS1 with its partners are summarized in Figure 2.

### 2.2. Functions of uS2 beyond the Ribosome

uS2 is a highly conserved r-protein essential for all organisms, from bacteria to humans, although its exact functions as a ribosomal component remain incompletely understood. It has been suggested that the prokaryotic uS2 might be involved in stabilizing the Shine-Dalgarno (SD) helix docked in a chamber between the head and the platform [78], as well as in protecting the SD duplex at the early post-initiation step [79]. However, this does not explain the vital function of uS2 in organisms that do not exploit the SD interactions in translation initiation. uS2 is one of the latest components in the 30S assembly [80]. In *E. coli* and most likely in other Gram-negative bacteria, its association with the 30S particle is indispensable for binding bS1, which accomplishes the assembly of the 30S subunit fully competent in recruiting mRNA [35,36]. Within the ribosome, uS2 is located on the back of the 30S subunit at the hinge between the head and body. Possessing an elongated bidomain structure, uS2 forms direct contacts with several 16S rRNA helices, viz, h35–h37 in the head via the coiled-coil α_2_ domain and h26 in the body via the large globular domain [81].

uS2 is encoded by the first gene of the *rpsB-tsf* operon that also comprises a gene for the elongation factor Ts (Figure 1). When synthesized in excess over the 30S ribosome, uS2 acts as a translational autogenous repressor of the *rpsB*-*tsf* mRNA [82,83]. As a repressor, uS2 recognizes and binds the unique structural features within the 5′UTR of the mRNA, inhibiting its own translation directly. This repression interrupts transcription–translation coupling in the operon, thereby decreasing the level of the bicistronic *rpsB-tsf* mRNA and hence the level of the essential Ts; that is why the expression of uS2 from a plasmid significantly slows down the growth rate [82].

The mRNA structural features recognized by uS2 are highly conserved, at least across γ-proteobacteria [83,84]. The mechanistic details of the autoregulation remain unclear as the ribosome binding site (RBS), including the SD sequence and the start codon, is not involved in the operator structure, and a small deletion of the conserved bulge far upstream RBS may eliminate the uS2-mediated regulation [82,83]. To act as an autogenous repressor effectively, uS2 needs a companion, bS1, with which it forms a complex not only on but also outside the ribosome [82], indicating an intimate relationship between these two r-proteins. Moreover, moderate overexpression of bS1 from a plasmid can suppress the thermosensitive phenotype of one of the *rpsB* mutants, *rpsB1^ts^*, allowing its growth at an elevated temperature otherwise lethal to the strain [85]. Thus, there are several distinct features of the uS2-mediated autogenous regulation as follows: (i) uS2 as an autogenous repressor is not a primary rRNA-binding protein but binds to the 30S subunit at the late step of the assembly; (ii) to serve as a repressor effectively, uS2 needs the assistance of bS1; (iii) the *rpsB* operator site bears no visible similarity to the regions on 16S rRNA bound by uS2 on the ribosome. However, more sophisticated analysis of the *rpsB* mRNA regulatory structure (in-cell PAIR-MaP analysis) has revealed a common architecture of the uS2 binding sites on 16S rRNA and the *rpsB* mRNA at least in enterobacteria [86].

Aside from its role as an autogenous repressor, uS2 may have other moonlighting activities in pathogenic bacteria, although the underlying mechanisms remain unclear and await further studies. Recently, RpsB (uS2) has been shown to be a surface-exposed protein of rickettsia, representing an important ligand and adhesin of these obligate intracellular microorganisms [87]. Increased expression of uS2-derived peptides has been observed in the highly virulent strains of *Streptococcus suis* cultured under host-simulated conditions, indicating that uS2 or its peptides might serve as specific virulence factors [88]. The role of uS2 in pathogenesis is obviously not related to its functions in the ribosome. The potential functioning of uS2 beyond the ribosome has also been proposed for a harmful human pathogen, *M. tuberculosis* (*Mtb*). *Mtb* RpsB has been identified not only in cytosolic but partially in cell wall fractions, while its counterpart in non-pathogenic *M. smegmatis* (*Msm*) is located only in the cytoplasm. Moreover, *Mtb* RpsB ectopically expressed in *Msm* has also been found to associate with the cell membrane/wall. *Msm* cells expressing *Mtb* RpsB in trans show reduced cell wall permeability and increased tolerance to drugs, oxidative stress, SDS, and starvation. An ability to impart stress resilience to mycobacteria can be ascribed to the unique C-terminal sequence of *Mtb* RpsB, which is absent from RpsB of non-tuberculosis mycobacteria, and the deletion of the C-terminal extra-fragment deprives *Mtb* S2 of its ability to influence resistance to stresses [89].

### 2.3. uS4, an Essential r-Protein Functioning in Ribosome Biogenesis, Translation, and Transcription

An essential r-protein, uS4, is a primary protein in the 30S ribosomal subunit biogenesis, which nucleates the assembly by binding to a five-way helix junction in the 16S rRNA 5′ domain. It is believed that early uS4–16S rRNA interactions guide rRNA folding and impact later steps of the 30S assembly [90,91,92]. Surprisingly, additional r-proteins, especially a late-binding protein, uS12, may accelerate the proper binding of uS4 during rRNA transcription by acting on the nascent rRNA as an RNA chaperonin [93]. Within the ribosome, uS4 is involved in multiple functions, including mRNA decoding, and mutations in uS4 have an impact on translation fidelity [94,95]. uS4, along with uS3 and uS5, is located at the mRNA entry site between the head and the shoulder of the 30S subunit and endows the ribosome with a helicase activity necessary to disrupt downstream helices in mRNA since the narrow mRNA channel is capable of accommodating only unpaired mRNA segments [96].

In addition to its role in ribosome biogenesis and the formation of the mRNA entry site, uS4 possesses moonlighting activities as a regulator of both translation and transcription. First, it is a regulatory protein in the post-transcriptional control of the α-operon that in *E. coli* comprises genes for four r-proteins and the α-subunit of RNA polymerase (uS13, uS11, uS4, RpoA, and bL17, in this order; see Figure 1). Autogenous repression of the *E. coli* α-operon mRNA translation by uS4 has been thoroughly studied for years [97,98,99,100,101]. Interaction of uS4 with the target site in the α-operon mRNA results in translational repression of not only the first three cistrons for uS13, uS11, and uS4 but also the last one encoding bL17, without affecting the intervening α-cistron that is regulated independently. A presumable second binding site for uS4 on the α mRNA in front of *rplQ* has been proposed, suggesting that uS4 may repress bL17 translation directly [102]; however, strong evidence for this has not been provided.

The operator site for the uS4-repressor on the *E. coli* α mRNA forms a complex pseudoknot structure comprising the ribosome binding site of the first cistron, *rpsM* [99,100,101]. The uS4 interaction with the pseudoknot traps mRNA in a conformation that allows binding of the 30S subunit but prevents the formation of the active initiation complex with the initiator tRNA, thus blocking translation. This inhibition mechanism is called “entrapment” to emphasize the lack of competition between the repressor and the ribosome for mRNA binding [100,101].

In *B. subtilus (Bsu)*, as well as in other species of the class Bacilli, the *rpsD* gene does not belong to the cluster of genes encoding uS13, uS11, α, or bL17; it is situated in a separate region of the chromosome, while the other genes are kept in the same order. At the same time, *Bsu* S4 binds to the untranslated leader of the *rpsD* mRNA and represses its own translation. The regulatory region does not form a pseudoknot, and hence, the regulation is essentially different from that in *E. coli* [103,104]. How the r-protein genes within the α operon in Bacilli are regulated remains unknown. Interestingly, the members of the class Clostridia, in contrast with the class Bacilli, keep the *rpsD* gene in the α operon (NCBI Gene Database). Although the *E. coli*-like gene order of the α operon containing *rpsD* is widely distributed in bacteria, there are many lineages where *rpsD* is separated. Thus, β-, γ-, and ε-proteobacteria bear an *E. coli*-like α operons, while in α-proteobacterial species, *rpsD* is located distantly (NCBI Gene Database). The reason for this diversity remains an open question.

**Figure 3:**
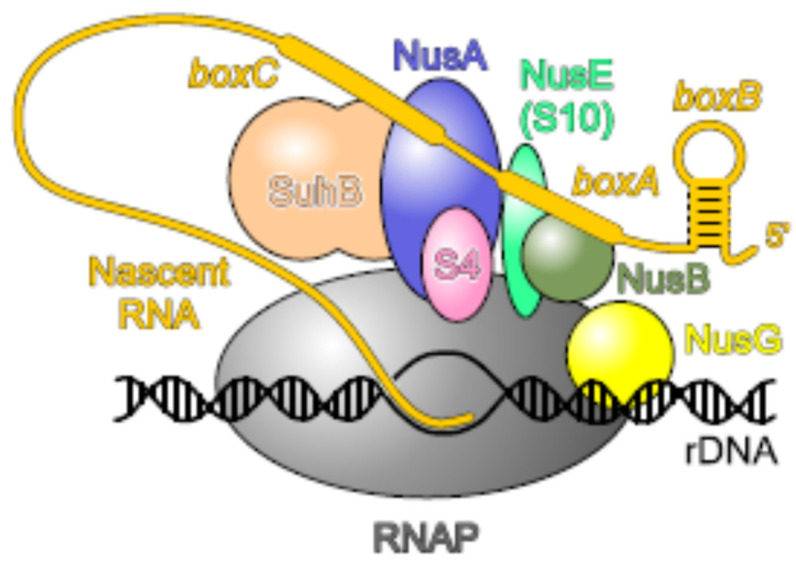
Composition of a specialized transcription complex to achieve efficient transcription of rRNA in *E. coli*. Two r-proteins moonlight as participants of the complex formation—uS4 and uS10 (NusE). The figure is adopted from [106].

Another well-known moonlighting activity of uS4 is its role in transcription as a general antitermination factor with properties very similar to NusA [105]. uS4 associates with RNA polymerase (RNAP) in vivo and inhibits the premature termination of the rRNA operons. The antitermination activity of uS4 is specific for Rho-dependent terminators. Thus, uS4, together with uS10 (NusE, see below), are important components of the *rrn* antitermination system involved in ribosome biogenesis. The antitermination complex is formed in response to *cis*-acting elements (boxB, boxA, and boxC) in the nascent pre-rRNA. Recent studies [106,107] show that Nus factors (A, B, E, and G), SuhB (the inositol mono-phosphatase), and uS4 assemble on RNAP into a capped ring around the RNA-exit channel, where uS4 serves as a flexible lid. Such a bulky protein structure may block an approach of the termination factor Rho to RNAP. Moreover, Nus factors, SuhB, and uS4 together support co-transcriptional rRNA folding by acting as an RNAP-associated RNA chaperone according to the well-known RNA-chaperone molecular principles [106,107]. The structure of the rRNA-specific antitermination complex is represented in Figure 3.

Finally, it has been recently found that uS4 (along with TufA and GacA) can be cross-linked in vivo in the stationary phase to a “mysterious” PA2504 protein from *Pseudomonas aeruginosa*. It was supposed that PA2504 might block the biological functions of these proteins to fine-tune the cellular response to stationary phase-dependent nutrient starvation [108]. It is currently unknown which of the uS4 functions is blocked by PA2504.

### 2.4. Ribosomal Proteins bS6 and bS18 Act in Tandem

Proteins bS6 and bS18 should be considered in tandem because they function as a heterodimer both in the ribosome assembly and in regulating the expression of their own operon *rpsF* (S6)-*priB*-*rpsR* (S18)-*rplI* (L9). In β- and γ-proteobacteria, this operon includes non-ribosomal gene *priB* that encodes the primosomal n protein necessary for replication restart, while in certain phyla, *priB* is not present in the operon (α-proteobacteria, Bacteroidetes/Chlorobi). Actinobacteria have *ssb* instead of *priB*, as well as Firmicutes, which, in addition, lack *rplI* at the end of the operon. Despite these differences, in most taxonomic groups, *rpsF* and *rpsR* are expressed from the same transcription unit and most likely are regulated jointly [109]. bS6 and bS18 are secondary binding proteins in the assembly of the central domain of a 30S subunit, which is nucleated by binding of the primary uS15 protein to a highly conserved 16S rRNA region. However, there are no protein–protein contacts between uS15 and bS6–bS18, and the rRNA site bound by bS18 within a heterodimer is formed due to the uS15-mediated structure remodeling [110].

Relatively recently, it has been discovered that bS6–bS18 proteins regulate the expression of their own operon at the translation level by binding to the 5′ UTR upstream of the *rpsF* start [109,111,112]. Initially, high phylogenetic conservation of a presumable regulatory region has been computationally predicted, and it has been demonstrated that a bS6–bS18 complex indeed binds to this RNA fragment from *E. coli* when in vitro. A putative RNA operator bears a conserved CCG sequence in a bulge flanked by a stem and a hairpin, which is analogous to the structural context of the 16S rRNA-binding site for bS6–bS18, thus suggesting the molecular basis for the autoregulatory mechanism [109]. Further, a wide distribution of the structural RNA motif in front of *rpsF* across many bacterial phyla has also been described by Meyer’s group, and the direct interaction of a bS6–bS18 complex with the RNA motif from *B. subtilis* has been confirmed [111]. Finally, in vivo, reporter experiments in *E. coli* have demonstrated that a bS6–bS18 complex indeed functions as an autogenous repressor to regulate expression of the operon by binding to the regulatory site preceding *rpsF*, with the bS18–mRNA interaction being crucial for the translation inhibition [112].

Interestingly, bS6 in *E. coli* is modified by the ATP-dependent glutamate ligase RimK, which can add up to four glutamate residues to the C-terminus of the protein. Oligoglutamylation of bS6 by RimK occurs only in the stationary phase [113]. It is yet difficult to ascribe any reasonable role for such a modification in *E. coli*. The same modification of bS6 by RimK has been studied for a soil bacterium *Pseudomonas fluorescence* [114,115], where it is able to change the expression of a set of genes encoding surface attachment factors, amino acid transporters, and secreted molecules. However, the mechanistic details of the effect of a single r-protein modification on gene expression and proteomic changes are not yet clear.

### 2.5. A Key Primary Assembly r-Protein uS7 Is Bifunctional

Ribosomal protein uS7 is a key primary protein in the 30S subunit assembly [80]. Its interaction with 16S rRNA initiates the folding of the 3′-major domain and further formation of the 30S head where it faces the decoding center. Like uS4, uS7 first forms numerous short-lived contacts with the 3′ domain of 16S rRNA, but the stable incorporation of uS7 is promoted by the secondary r-proteins uS9, uS13, and uS19, which act as chaperones to provide correct folding of the rRNA helices [92]. Aside from the interaction with 16S rRNA, uS7 forms contacts with uS9 and uS11 within the ribosome [81].

uS7 stably associates with the trigger factor chaperone (TF) in vivo in *E. coli*, in *Thermotoga maritima* [116], and likely in other bacteria [117]. TF in the TF:S7 complex masks 16S rRNA binding sites on uS7, and uS7 within the complex is more stable than free uS7 in solution. It was suggested that by providing the correct folding of r-proteins (e.g., uS7), TF might act as a ribosome assembly factor [117].

The only moonlighting activity of uS7 described so far is its ability to serve as an autogenous translational repressor of the *str* operon. If uS7 synthesis in a cell exceeds synthesis of 16S rRNA, the same RNA-binding determinants that provide uS7 binding to 16S rRNA participate in the binding of uS7 to its own *str* mRNA [118,119]. The *str* operon encodes r-proteins uS12 and uS7 and the translation elongation factors EF-G (*fus*) and EF-Tu (*tufA*) in this order (Figure 1). To inhibit translation of the *str* mRNA, uS7 binds to the intercistronic region preceding its own cistron [118,119,120,121]. Interestingly, uS7 acts as a translational repressor in vivo only in the presence of the intact *rpsL* (uS12) cistron but does not repress independent *rpsG* (S7) translation, indicating that the coupled *rpsL-rpsG* translation is indispensable to achieve autogenous repression [120]. Both targets, 16S rRNA and *str* mRNA, bear similar sites recognized by uS7 [119,121]. Overexpression of uS7 from a plasmid inhibits bacterial growth due to repression of the essential *fus* gene (EF-G), whose translation is coupled with *rpsG* [119]. At the same time, expression of the last gene in the *str* operon, *tufA*, that encodes EF-Tu, is not noticeably inhibited because of the presence of two additional promoters within the *fus* gene (Figure 1). The first cistron, *rpsL*, is regulated by the “retroregulation” mechanism based on the destabilization of the corresponding mRNA region by the repressor binding [120].

A similar mechanism likely regulates the *str* operon in cyanobacteria where the intercistronic region separated *rpsL* and *rpsG* bears structural similarity with the S7-binding region of 16S rRNA [122]. It is a pity that the uS7-mediated regulation has not been studied in other bacterial phyla since it has been reported that while extended distances between the uS12 and uS7 cistrons exist in many species, the mRNA structure observed in *E. coli* is not obviously conserved [84].

### 2.6. uS8 Regulates the Longest spc Operon

uS8 is an important rRNA-binding protein that occupies a central position within a 30S subunit. It interacts with 16S rRNA specifically by binding the helix h21 and is crucial for the correct folding of the central domain of 16S rRNA [81]. The binding of uS8 to 16S rRNA has been extensively characterized using a variety of techniques. A minimal 16S rRNA fragment located in helix 21 was shown to be sufficient for the specificity and high affinity of the uS8–rRNA interaction [123].

Comparable to uS7, uS8 is bifunctional. It serves as an autogenous repressor controlling the translation of the *spc* mRNA [124,125,126,127]. The *spc* operon in *E. coli* is the longest r-protein operon encoding uL14, uL24, uL5, uS14, uS8, uL6, uL18, uS5, uL30, and uL15, and, in addition, comprises the *secY* gene encoding a component of the protein export machinery, and *rpmJ*, a gene for a small r-protein, bL36 (Figure 1). The regulatory mechanism is analogous to the repression of the *str* operon by uS7 (see above). The repressor uS8 binds not upstream of the first cistron but at the initiation region of the third cistron, *rplE*, encoding uL5. This binding directly blocks the translation of *rplE*, while the translation of the downstream cistrons appears inhibited due to the interruption of translational coupling [126]. The first two cistrons, *rplN* and *rplX*, are subject to “retroregulation” resulting from the mRNA destabilization [127]. Regulation of the last two cistrons, *secY* and *rpmJ*, remains unclear.

The S8 binding site at the beginning of *rplE* (uL5) is very similar to the S8 binding site on 16S rRNA [123,125,128,129]. The structure of the complex of uS8 with its operator site on the *spc* mRNA has been resolved with high resolution, and it has been shown that uS8 uses the same RNA-binding site both for 16S rRNA and mRNA binding [128,129]. The high similarity of both uS8 RNA targets implies the principle of molecular mimicry. The *spc* operon of *V. cholerae* (γ-proteobacterium) is autogenously regulated by uS8, presumably in an *E. coli*-like manner [130], but how the *spc* operon is regulated in *B. subtilis* or other species remains unknown [131]. Given that the *E. coli*-like structure involved in uS8 binding with the *spc* mRNA is not found in *B. subtilis*, the regulatory mechanism seems to be different [131]. Unfortunately, phylogenetic studies of the *spc* mRNA autogenous regulation have not been advanced.

### 2.7. uS10, an Essential Player in Transcription–Translation Coupling and Transcription Antitermination

uS10 is a tertiary binding protein in the 30S assembly; its addition to the assembly intermediates depends on uS9, a secondary protein, and uS7, a primary assembly protein interacting with 16S rRNA [132]. A well-studied functional role of uS10 in *E. coli* ribosomes is its assistance in transcription–translation coupling, where uS10 provides physical contacts between the leading ribosome and RNA polymerase (RNAP) synthesizing the mRNA [133,134]. Another factor critical for a direct link between RNAP and a translating ribosome is NusG. NusG contacts with RNAP via its N-terminal domain, while through its C-terminal domain, it can physically interact with uS10 on the 70S ribosome or with the termination factor Rho to stimulate Rho-dependent termination [134,135]. A competition between uS10 and Rho for NusG may explain why Rho cannot terminate translated transcripts. When the translation rate matches that of transcription, an approach of Rho to the transcript is blocked by the NusG interaction with uS10 on the ribosome, but when translation is inhibited, weakened NusG–uS10 contacts lead to uncoupling, resulting in RNAP backtracking and Rho-mediated termination [136,137]. Close relations between the leading translating ribosome and transcribing RNAP have led to the suggestion that they may form a physical complex, a so-called ‘expressome’, a molecular super-machine performing both steps of gene expression [138,139,140]. However, the current models structurally describing bacterial transcription–translation coupling are rather controversial [140].

Surprisingly, in contrast with *E. coli*, transcription and translation in *B. subtilis* seem to be functionally uncoupled, and an active transcription elongation complex may be independent of the leading ribosome, suggesting that *E. coli* and *B. subtilis* use divergent regulatory mechanisms [141]. While translation elongation in these species proceeds with a similar rate, the transcription elongation rate of mRNAs in *B. subtilis* is nearly twice as fast as that in *E. coli* (runaway transcription). Moreover, factors that mediate translation–transcription coupling, Rho and NusG, are essential in *E. coli* but dispensable in *B. subtilis*. Phylogenetic analysis predicts that the uncoupling of transcription and translation may be widespread in Gram-positive bacteria [141]. Thus, the idea that transcription and translation are tightly coupled in all bacteria appears misleading, i.e., in *E. coli*—tightly coupled; in *Bacillus*—uncoupled; in other bacteria—we do not know yet.

The most studied moonlighting activity of uS10 is its functioning in the antitermination of transcription, first discovered in studies of phage λ. Upon transcription of N-utilization (*nut*) sites in the λ genome, the phage protein λN and a set of host Nus factors (N-utilization substances) A, B, E (uS10), and G associate with RNAP, thus enabling the enzyme to read through intrinsic and Rho-dependent terminators [142]. uS10 is the first r-protein that has been shown to participate in transcription regulation [143]. It forms a complex with NusB to bind to a single-stranded boxA motif on λ *nut* sites. The same boxA motif is present in the *E. coli* rRNA operon (*rrn*) transcripts, and binding of uS10–NusB to the boxA sequences in the nascent rRNA is indispensable for *rrn* antitermination. The bound to boxA uS10–NusB complex interacts with elongating RNAP via the uS10–NusG interaction [144,145]. It should be noted that in the complex with NusB, uS10 adopts the same fold as in the 30S subunit and is blocked from simultaneous association with the ribosome [146]. Thus, the functions of uS10 in transcription–translation coupling and in antitermination are very similar: in both processes, its interaction with NusG bound to RNAP prevents the Rho-dependent transcription termination.

Further studies have identified an additional member of the *rrn* antitermination machinery, SuhB (inositol mono-phosphatase), and revealed that a complex of Nus factors (NusB, NusE, NusA, NusG, and SuhB) not only participates in antitermination on the *rrn* operons but also ensures correct folding and maturation of rRNA [147]. Moreover, Nus factors may act beyond rRNA and regulate the expression of mRNAs as well. Thus, in *E. coli*, binding of the NusB–NusE (uS10) complex to the boxA sequence within the *suhB* 5′UTR represses translation of the *suhB* mRNA. This binding sterically prevents the ribosome from initiating translation, which in turn promotes Rho-dependent termination within the *suhB* gene due to transcription–translation uncoupling [148]. It is believed that the boxA-mediated regulation of the Nus factors is highly conserved and widespread.

The regulatory activity of the Nus factors has been recently proposed to be involved in the complex regulatory cascade of flagella biosynthesis [149]. One of the flagella-specific sRNAs implicated in the regulation of flagellar operons, MotR, appears to base pair internal to the *rpsJ* (uS10) coding sequence and promote Hfq binding to the *rpsJ* leader sequence, which in turn results in increased *rpsJ* translation. Due to its elevated concentration in the cell, uS10, in conjunction with NusB, may increase transcription antitermination of long flagellar operons, contributing to flagellin protein levels, flagella numbers, and cell motility [149].

### 2.8. uS15, a Translational Auto-Repressor in Various Bacterial Phyla

uS15 is a primary protein in the 30S assembly pathway. It interacts with a highly evolutionary conserved central domain of 16S rRNA, comprising a three-helix junction (h20, h21, h22), and this interaction is required for the subsequent binding of other proteins (e.g., bS6, bS18) necessary for the formation of the 30S subunit platform [110]. Surprisingly, despite its primary role in the 30S assembly, uS15 is not essential, as the strains with a deleted *rpsO* gene are viable, albeit reveal a cold-sensitive phenotype [150]. This means that under appropriate temperature conditions in vivo, the 30S assembly may proceed in the absence of uS15.

The only extraribosomal activity of uS15 found so far is its functioning as an autogenous repressor of the *rpsO* gene. The uS15-mediated autogenous control represents the most studied case among the regulatory processes involving r-proteins; it has been examined in various bacterial species, including *E. coli* [151,152,153,154,155,156], *B. stearothermophilus* [157,158], *Geobacillus kaustophilus* [159], *Thermus thermophilus* [160], and *Rhizobium radiobacter* [161]. In all these cases, the autoregulation operates at the translation initiation level but through different mechanisms, e.g., in *E. coli*, binding of uS15 to the *rpsO* mRNA leads to the ribosome ‘entrapment’ in a non-productive complex [151], while direct competition with the ribosome binding occurs in *T. thermophilus* [160] and *B. stearothermophilus* [157,158]. In contrast to the high conservation of uS15 and its 16S rRNA targets, the regulatory structures on the *rpsO* mRNAs vary widely both at the primary and secondary structure levels, which suggests the presence of many ways to allow autogenous regulation [159,161,162].

In *E. coli*, the regulatory site (operator) on the *rpsO* mRNA folds in a pseudoknot that is stabilized by uS15, allowing the 30S ribosome to bind but preventing the formation of an active initiation complex [151,152,154,155]. The only common determinant shared by the two uS15 targets on rRNA and mRNA is a U-G/C-G motif that contributes modestly to rRNA binding but is crucial for mRNA recognition [153,154,155]. Unlike *E. coli*, the operator structures for uS15 on the *B. stearothermophilus* and *T. thermophilus rpsO* mRNAs are organized in three-way junction motifs that mimic the conserved three-way junction of the S15 rRNA-binding site [157,158,160]. Stabilization of the three-helix junction on the mRNA by uS15 may prevent ribosome binding, thereby blocking translation initiation.

**Figure 4:**
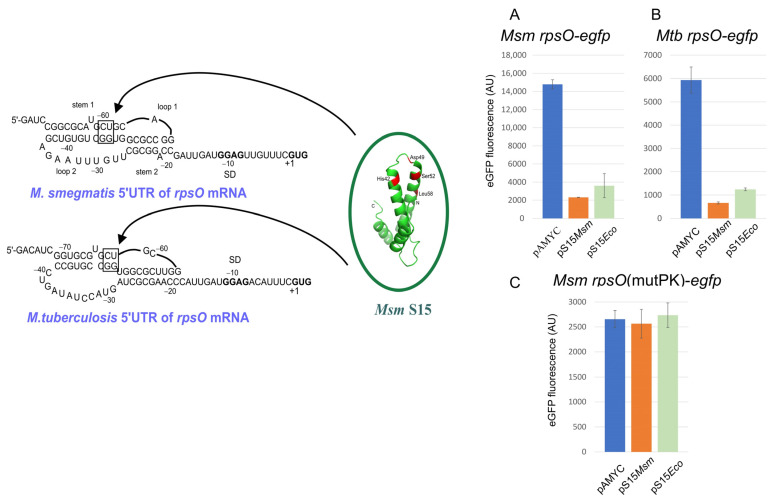
Mycobacterial uS15 represses its own translation by binding to the pseudoknot structure within the *rpsO* 5′UTR [163]. **On the left**: Predicted secondary structures for the *Msm* and *Msm rpsO* 5′UTRs form pseudoknots. Initiator codons and SD sequences are in bold; conserved U-G/C-G motifs recognized by uS15 are framed. Three-dimensional structure of free *Msm* uS15 is enclosed in green oval. **On the right:** (**A**,**B**) Results of in vivo fluorescent reporter assays for *M. smegmatis* (*Msm*) and *M. tuberculosis* (*Mtb*) *rpsO*-*egfp* fusions incorporated in the *Msm* chromosome. pAMYC—an empty vector; pS15*Msm* and pS15*Eco*—pAMYC derivatives expressing uS15 from *Msm* and *E. coli*. (**C**) uS15-mediated repression disappears after pseudoknot mutagenesis (mutPK).

Recently, we have examined the *rpsO* regulation in mycobacteria *M. smegmatis* (*Msm*) and *M. tuberculosis* (*Mtb*) and provided evidence for the S15-mediated autoregulation at the translation initiation level [163]. Remarkably, the autogenous regulation of the mycobacterial *rpsO* genes appears to strictly require the pseudoknot conformation of the 5′UTR so that mutations disrupting the pseudoknot completely abolish the uS15-mediated translational repression (Figure 4). As in the case of *E. coli*, a U-G/C-G motif in a pseudoknot turns out to be crucial for the autogenous control. Moreover, *E. coli* S15 appears capable of acting as an efficient repressor of the *Msm/Mtb rpsO* expression, but this ability has been lost after destroying the pseudoknot. Thus, the mechanism for the uS15-mediated autogenous control in mycobacteria is very similar to that described for *E. coli* despite the large phylogenetic distance between these species. At the same time, while the regulatory pseudoknot in *E. coli* embraces the *rpsO* SD sequence and the initiator codon (in a loop2 region), the pseudoknots in mycobacteria are situated upstream from the initiation site, (Figure 4) implying that the mechanistic details of the autogenous repression may be different, and in mycobacteria, uS15 binding to the pseudoknot might prevent ribosome binding rather than entrap the ribosome.

### 2.9. bS20, a Curious Case of a Regulatory Protein

bS20 is one of the six primary r-proteins (along with uS4, uS7, uS8, uS15, and uS17) that bind to 16S rRNA during the 30S subunit assembly. bS20 may interact with at least two regions on 16S rRNA, in the 5′domain and the 3′minor domain (specifically, with the helix 44), bringing these very distant regions into proximity [164,165]. This is a puzzle because, in vivo, the 3′domain is transcribed much later than the 5′domain, given that the 30S ribosome assembly proceeds co-transcriptionally.

bS20 is encoded by the gene *rpsT*, which is a monocistronic operon located apart from clusters of most r-protein genes on a bacterial chromosome (Figure 1). Though bS20 is a primary binding protein, it is not essential, but its absence results in slow growth due to a poor assembly of the 70S initiation complex and defects in the translation initiation. These defects are caused by a significant reduction in the rate of mRNA association rather than an impairment in P-site fMet-tRNA^fMet^ binding [166]. Curiously enough, being a 30S subunit r-protein, bS20 has been repeatedly co-purified with the 50S subunit and even designated as L26. Moreover, the copy number of bS20/bL26 in the 70S ribosome was evaluated as 1.38, thus implying the possibility of its independent binding to each subunit [167]. 

A specific feature of the *rpsT* mRNA is the use of the otherwise inefficient UUG as a start codon, which is unusual for the mRNA of a highly abundant r-protein. The use of UUG is not widespread among bacterial *rpsT* mRNAs, being typical only for several γ-proteobacterial families (e.g., Enterobacteriaceae, Pasteurellaceae, and Vibrionaceae) but not for others, e.g., members of Pseudomonadaceae, Legionellaceae, and Xanthomonadaceae use GUG, and alpha-, beta-, and epsilon-proteobacteria use a normal AUG start codon to initiate translation of the *rpsT* mRNAs (NCBI Gene Database).

It is believed that, like other primary r-proteins in *E. coli*, bS20 may regulate its own synthesis as an autogenous repressor, and the weak start codon plays a fundamental role in the autoregulation, allowing bS20 to compete with ribosomes for mRNA binding [168]. However, compelling arguments have not been provided, and attempts to demonstrate any measurable affinity of bS20 for its own mRNA have appeared unsuccessful [169]. No conserved mRNA secondary structures typical of autogenous operators for r-protein-repressors have been found either [84]. Thus, until now, the belief that bS20 may act as a translational autogenous repressor has neither been confirmed nor refuted.

### 2.10. bS21 and Heterogeneity of Ribosome Population

In *E. coli*, the *rpsU* gene encoding bS21 belongs to the unique operon called the macromolecular synthesis operon (MMS), which comprises genes involved in the initiation of the major processes in the flow of genetic information as follows [170]: bS21 (*rpsU)* in the initiation of translation; DNA primase (*dnaG)* in the initiation of chromosome replication; and the *rpoD*-encoded major sigma factor, sigma-70, in transcription initiation (Figure 1). *E. coli* bS21 is an essential r-protein that participates in translation initiation by providing the base-pairing of the 3′ terminus of 16S rRNA with the SD sequence on mRNA [171]. It should be noted that *rpsU* is part of the MMS operon only in Gram-negative relatives of *E. coli*, while in Gram-positive Firmicutes, *rpsU* is situated separately (NCBI Gene Database). A striking example is the absence of bS21 in every member of Actinobacteria. This protein is also missing in all representatives of the phyla Deinococcus-Thermus, Fusobacteria, and Thermotogae [23]. Conversely, some bacterial species encode multiple bS21 homologs, e.g., an intracellular bacterial pathogen, *Francisella tularensis*, encodes three distinct homologs of bS21 [172,173].

bS21 is one of the last proteins in the 30S assembly, which is loosely bound to and easily exchangeable among ribosomes [174]. Its absence in a part of the cellular ribosomal population leads to intrinsic ribosome heterogeneity and, hence, may provide a regulatory capacity. The presence of several bS21 homologs, as in *F. tularensis*, also implies a ribosome heterogeneity that can contribute to the post-transcriptional regulation of gene expression. Recent data convincingly demonstrate how variations in the bS21 content may affect the translation efficiency of certain mRNAs.

Selective translational control mediated by bS21 has been reported for *Flavobacterium johnsoniae* [175,176]. Representatives of Flavobacteria, as well as other members of the phylum Bacteroidota, do not use the SD interactions for translation initiation. Although the anti-SD sequence is present at the 3′-terminus of 16S rRNA, it is buried in a pocket formed by bS21, bS6, and bS18 on the 30S platform and, hence, is unavailable for base-pairing with mRNA. The C-terminal region of bS21, highly conserved in Bacteroidota but not in other phyla like γ-Proteobacteria, is responsible for the anti-SD sequence sequestration [175]. The *rpsU* mRNA in Flavobacteria represents an exception in that it bears unusually extended Shine-Dalgarno sequences and, therefore, can be efficiently translated only by a subpopulation of ribosomes lacking bS21, resulting in replenishing the cellular amount of bS21 [176]. This kind of autoregulatory mechanism represents a unique case when the r-protein serves as an autogenous translational regulator not in a free state but as an integral part of the ribosome. It should be mentioned that *rpsU* regulation in *E. coli*, unlike Flavobacteria, remains unknown.

The presence of several bS21 homologs in the cell can also play a regulatory role. In a human pathogen, *F. tularensis*, one of the three bS21 homologs, bS21-2, specifically governs the translation of virulence genes [172]. The mRNAs responsive to bS21-2 bear specific features in their 5′UTRs, such as an imperfect SD sequence and a particular six-nucleotide sequence, while mRNAs with a classic SD element do not require bS21-2 for translation [173]. This raises the possibility that other bS21 homologs in *F. tularensis* or other organisms may influence translation in a leader sequence-dependent manner.

## 3. Multifunctional Proteins of the 50S Ribosomal Subunit

### 3.1. uL1 as a Widespread Autogenous Repressor

uL1 is a highly conserved two-domain protein that binds 23S rRNA helices H76 to H78 with the formation of the so-called L1-stalk. This mobile structural element governs tRNA dynamics during translation elongation and is responsible for the release of deacylated tRNAs from the ribosomal E-site [177,178,179]. In most bacteria, the *rplA* gene encoding uL1 is co-transcribed with *rplK* that encodes uL11 (Figure 1). It has been revealed that uL1 is bifunctional and uses its prominent RNA-binding properties to autogenously regulate the *rplK*-*rplA* expression at the translation level [180,181]. Moreover, the uL1-mediated autogenous regulation has been observed not only in bacteria but also in Archaea, and due to the high evolutionary conservation, bacterial L1 proteins are able to regulate archaeal L1-specific mRNAs [182,183]. The L1-binding sites on the mRNAs are very similar, both in sequence and secondary structure, to the uL1 binding site on 23S rRNA, indicating the principle of molecular mimicry underlying autoregulation [183]. At the same time, the complex of uL1 with 23S rRNA is more stable than the regulatory complex with the mRNA of the L11-L1 operon, so the appearance of a newly synthesized rRNA in the cell releases uL1 from the repressor complex [183].

The crystal structure has been resolved for several uL1 homologs from bacterial and archaeal species [184,185]. The proteins consist of two domains, with domain I including the N- and C-termini, and domain II corresponding to the central part. This structure implies that domain II represents an insertion in domain I and can be deleted, which is useful for studying the role of each domain. Several studies show that RNA-binding properties belong to domain I, and domain II just stabilizes the uL1–RNA complex. Moreover, the isolated domain I from *T. thermophilus* uL1 can be incorporated in vivo in the *E. coli* and *T. thermophilus* ribosomes [186,187] and possesses regulatory activities in vitro similar to the intact protein [187,188].

The binding sites for the uL1-repressor on the mRNAs are situated in the 5′UTR preceding the L11 (*rplK*) cistron (in Proteobacteria) or in a region preceding the L1 cistron (Actinobacteria, Cyanobacteria), or, in some cases, there exist two L1-binding sites, one upstream of *rplK*, another upstream of *rplA* (e.g., in Firmicutes) [84]. In all cases, the regulatory site consists of an irregular stem-loop structure with an internal bulge [84]. Recent data have demonstrated that thermophilic bacteria *T. thermophilus* and *T. maritima* also bear two uL1-regulatory sites preceding each gene in the *rplK-rplA* operon [188]. This might be necessary to provide enhanced regulation of gene expression in the organisms living at high temperatures.

### 3.2. Multiple Activities of uL2

Ribosomal protein uL2 is encoded in the long *S10* operon regulated by uL4 (Figure 1). It plays important roles in the assembly of the 50S ribosomal subunit (by incorporating at the early stages of biogenesis), binding of tRNA to the A and P sites, the peptidyl transferase activity of the 50S subunits, and peptide bond formation [189]. uL2 is thought to be one of the most evolutionarily ancient and conserved proteins of the large ribosomal subunit [190]. uL2 contains a solvent-accessible globular domain that contacts the 30S subunit through bS20 and the stem regions of helices h23 and h24, thus forming the intersubunit bridge B7b [189,191]. Although it has been suggested that peptidyl transferase activity is primarily a property of rRNA [9,11] and r-proteins may act only as scaffolding, accumulating evidence has shown that uL2 is functionally essential for the peptidyl transferase center (PTC) [189,192,193]. uL2 interacts with PTC through its C-terminal domain, and mutations in this domain confer resistance to the antibiotic bactobolin that specifically inhibits the peptidyl transfer step of translation [194]. Thus, uL2 represents a unique site directly targeted by the antibiotic capable of blocking PTC. At the same time, mutations in uL2 do not confer resistance to other PTC-inhibiting drugs like chloramphenicol, clindamycin, or linezolid [194].

The RNA-binding domain of uL2 is homologous to the OB-fold [193]. It is of interest that in T4-infected *E. coli* cells, uL2, similarly to bS1, is ‘RNAylated’ by the T4-encoded adenosine diphosphate-ribosyltransferase ModB that covalently attaches NAD-capped RNAs to defined arginine residues of the OB-fold proteins [77]. Future studies will reveal how the ‘RNAylation’ of uL2 and bS1 can affect the translation efficiency of the ribosome in T4-infected cells.

In addition to its essential functions as a ribosomal component, uL2 possesses several moonlighting activities. First, uL2 plays a specific and direct role in transcription, both in vitro and in vivo, acting as a transcriptional modulator through its interaction with the RNAP α-subunit in *E. coli*. Associated with the α-subunit, uL2 can specifically increase the activity of the P1 promoter of rRNA operons, thereby contributing to the coordination of the synthesis of ribosomal components [195]. Further, uL2 has been suggested to act as an inhibitor of the *oriC* unwinding by DnaA and the assembly of the *oriC* prepriming complex. As reported, uL2 or its truncated form lacking 59 C-terminal amino acid residues may physically interact with the N-terminal region of DnaA to inhibit the initiation of replication on *oriC* plasmids. This activity of uL2 may serve to coordinate the initiation of DNA replication with cell growth [196]. The moonlighting activities of uL2 also include its recently discovered interaction with a novel sRNA23 involved in the regulation of pathogenicity in *Streptococcus suis*. However, the exact role of *u*L2 in bacterial pathogenesis is yet to be explored [197].

### 3.3. uL4 Is Multifunctional

*E. coli* uL4 is an important r-protein that participates in the assembly of the 50S subunit and its proper functioning so that the uL4 mutant ribosomes are compromised in multiple steps of protein synthesis. Moreover, the uL4 alteration has numerous effects on the structure and function of both 50S and 30S subunits [198]. Structurally, uL4 consists of a globular domain residing on the 50S subunit surface and an extended loop (“tentacle”) that penetrates the core where it forms multiple contacts with 23S rRNA in the peptide exit tunnel in the vicinity of PTC. This is a place where erythromycin and other macrolides bind, and mutations in the uL4 loop strongly reduce ribosome susceptibility to these antibiotics (Ref. [199] and references therein). At the same time, there are no direct contacts between the uL4 loop and erythromycin, and resistance is most likely caused by perturbations in the 23S rRNA structure due to the uL4 loop mutations, leading to the narrowing of the tunnel entrance site and sterically impeding erythromycin binding [200].

Beyond the ribosome, uL4 possesses several activities due to its RNA- and protein- binding features. First, uL4 is a specific regulator controlling the expression of its own operon, *S10* (Figure 1). Unlike many other r-proteins acting as autogenous translational repressors, uL4 regulates both the transcription and translation of the *S10* operon [22]. In both cases, uL4 binds within a long, highly structured 5′UTR of the *S10* operon mRNA, with the regulatory sites for transcription and translation regulation partially overlapping. Transcription inhibition is believed to be caused by premature transcription termination at a Rho-independent termination site in the leader sequence. The mechanism most likely includes a transcription factor NusA that promotes RNAP pausing at an attenuator hairpin, as well as further stabilization of the paused transcription complex by uL4 binding to the upstream elements [201,202]. uL4 binding to the mRNA leader, in vitro, is outcompeted by the 23S RNA fragment comprising the uL4 binding site within the ribosome, thus implying the structural similarity of the two RNA targets for uL4 [203]. In contrast to the high phylogenetic conservation of uL4, its regulatory site on the *S10* mRNA is not widely conserved, even in γ-proteobacteria—structural and functional conservation were shown for members of enterobacteria, Haemophilus influenzae, and Vibrio cholerae, but not for *Pseudomonas*, suggesting that the *E. coli*-like uL4-mediated regulatory mechanism has emerged rather lately during the γ-proteobacterial evolution [204].

In addition to the RNA-binding sites involved in interactions with rRNA and mRNA, uL4 contains a C-terminal protein-binding interface, which is potentially able to form protein–protein contacts [205]. As reported later, uL4 indeed forms such contacts, both on the ribosome and beyond [206,207,208]. On the ribosome, uL4 interacts with the DEAD-box RNA helicase SrmB that is involved inthe early steps of the 50S subunit assembly [206,207]. Beyond the ribosome, uL4 binds to the C-terminal region of RNase E, outside of its catalytic domain, thereby stabilizing mRNAs targeted by RNase E in vivo. This interaction is involved in controlling plasmid DNA replication by stabilizing an antisense regulatory RNA normally attacked by RNase E. Inhibiting by ectopic expression of uL4 of the RNase E activity towards a set of mRNAs for stress-responsive proteins is believed to account, at least in part, for the bacterial adaptation to adverse conditions [208].

One more moonlighting activity of uL4 has been recently discovered as unrelated to its protein–protein interaction with RNase E [209]. uL4 appears to fine-tune the level of TnaA protein (tryptophanase) independently of RNase E inhibition. Post-transcriptional uL4-mediated regulation of the *tnaCAB* operon expression is likely based on uL4 binding to the operon mRNA within the spacer between *tnaC* and *tnaA*, which leads to the alteration of the spacer structural conformation and, as a result, downregulation of translation of the *tnaA* cistron [209]. The ability of the ribosomal protein to repress non-ribosomal mRNAs is a rare case, and sequence/structure features underlying the impact of uL4 on the *tnaA* translation deserve further investigation. 

### 3.4. bL7/12 and uL10 Form Pentameric Complex, Both on and beyond the Ribosome

Ribosomal protein bL12 (bL7/L12) is the only multi-copy r-protein, thus representing an exception to the rule of equal molarity of ribosomal components. 50S subunits of bacterial ribosomes may comprise two (like in *E. coli*), three (like in *T. maritima* or *M. smegmatis*), or even four dimers of bL12, as in some cyanobacteria, with the bL12 copy number being independent of the living conditions of bacteria [210]. The dimers of bL12 form a stable complex with uL10, termed the L7/12 stalk, which interacts with uL11 and 23S rRNA in the 50S subunit structure [211]. The L7/L12 stalk serves to recruit the translation initiation, elongation, and termination factors (IF2, EF-G, EF-Tu, RF3, and LepA) by the ribosome in their GTP states and, thereby, is critical for the translation processes [212,213,214,215]. The common interaction site for translational GTPases is the C-terminal domain of bL12 [214].

The pentameric complex L10(L12)_4_ participates in the autogenous regulation of the *rplJ-rplL* mRNA at the level of translation initiation [216]. The *rplJ* and *rplL* genes are promoter-proximal genes of the *rif* operon that also comprise the *rpoB* and *rpoC* genes encoding β and β’ subunits of RNAP (Figure 1). The expression of *rpoBC* is regulated independently by the autogenous control mechanism [217]. Autogenous regulation of the *rplJ-rplL* mRNA by a pentameric complex of its products provided the first evidence that r-proteins can act as repressors not individually but in concert with their partners in the ribosome structure. Subsequently, the same type of regulation has been demonstrated for the bS6–bS18 and uS2–bS1 regulatory complexes (see above, Section 2.2 and Section 2.4). A key role in the autogenous regulation of the *rplJ-rplL* mRNA belongs to uL10, which recognizes similar features on 23S rRNA and the leader sequence of the mRNA. In both cases, the RNA targets comprise a ‘kink-turn’ structural motif [218]. Mutations introduced in analogous positions of the kink-turn motifs on rRNA and mRNA have affected the corresponding RNA–protein interactions in a similar way, thus providing a strong argument in favor of a high similarity of the uL10 recognition sites [216,218]. The detailed mechanism of the translational autorepression in *E. coli* has not yet been resolved, as the recognition site on the 5′UTR is located rather distantly from the ribosome binding site of the *rplJ* cistron, making direct competition between the repressor and the initiating ribosome unlikely.

Autogenous regulation by a pentameric L10(L12)_4_ complex has also been demonstrated in *B. subtilis*, but the underlying mechanism turns out to be principally different from that in *E. coli* [219]. Like in the case of bL20 (see below), the *rplJL* autoregulation in *B. subtilis* occurs not at the translational level but at the level of transcription through transcription attenuation. The long 5′UTR of the *B. subtilis rplJL* mRNA can be folded in structures that function as an anti-antiterminator, antiterminator, or intrinsic terminator. The model proposed specifies that a pentameric L10(L12)_4_ complex binds to and stabilizes the anti-antiterminator structure, comprising a kink-turn motif, thus promoting transcriptional termination [219]. Most likely, this mechanism is highly conserved across *Bacillus* species. The regulatory mRNA region for a pentameric L10(L12)_4_ complex is widely phylogenetically distributed and may be present in more than half of the sequenced Fusobacteria, Actinobacteria, Cyanobacteria, and Chloroflexi [84]. However, the mechanisms underlying the regulation might be divergent and should be experimentally explored.

### 3.5. Ribosomal and Extraribosomal Functions of bL9

Though nonessential, the 23S rRNA-binding r-protein bL9 has been reported to play an important role in reading-frame maintenance in *Salmonella enterica* [220], ribosomal “hopping” over a 50-nucleotide region within the mRNA of the bacteriophage T4 gene 60 in *E*. *coli* [221], and response to starvation stress [222,223]. *E. coli* bL9 is stably phosphorylated, with all phosphorylation sites being located at the carboxyl-terminal domain (CTD). Phosphorylation of bL9 causes a complete disordering of its CTD and helps cell survival under nutrient-limiting conditions. It has been suggested that the conformation of the bL9 CTD may be involved in regulating the RelA function [223]. Another important role for bL9 has been proposed, owing to mapping the intracellular organization of translating ribosomes in *Mycoplasma pneumoniae*. It has been shown that their association with polysomes involves a local coordination mechanism mediated by bL9. The model implies that an extended conformation of bL9 within polysomes helps to maintain translation fidelity by avoiding direct collision within polysomes during active translation elongation [224].

The abovementioned activities are related to bL9 within the ribosome. Recently, an unforeseen moonlighting activity of bL9 has been revealed [225]. In *P. aeruginosa*, bL9 appears to repress translation of the *exsA* mRNA by binding to its 5′ UTR. No obvious sequence similarity exists between 5′UTR of the *exsA* mRNA and the 23S rRNA region involved in bL9 binding during the ribosome assembly. ExsA is a master regulator that activates the transcription of all genes of the type III secretion system (T3SS), a critical virulence determinant of *P. aeruginosa*. Therefore, by inhibiting the *exsA* translation, bL9 serves as a novel T3SS repressor. This finding represents a rare case when the r-protein can regulate the translation of the non-ribosomal mRNA.

### 3.6. uL13, a Novel Autogenous Repressor

An essential r-protein, uL13 is an early 50S assembly component that interacts with 23S rRNA. Its incorporation in vivo requires a DEAD-box RNA helicase SrmB that is required to organize the uL13 binding site on 23S rRNA by preventing the formation of improper alternative structures [207,226]. uL13 is encoded by a promoter-proximal gene of the operon *rplM-rpsI* (uL13-uS9) (Figure 1). Regulation of the uL13-uS9 operon has been recently studied at the transcriptional and translational levels [27]. Transcription of *rplM-rpsI* is subject to negative stringent control, as in the case of many other ribosomal operons, while its translation is autogenously regulated by uL13, which serves as a highly specific translational repressor of both *rplM* and *rpsI* expression if produced in excess over 23S rRNA available for de novo ribosome assembly [27]. To act as a translational repressor, uL13 binds to a highly structured 5′UTR of the operon mRNA. This 157-nt-long 5′UTR folds in a developed secondary structure that comprises several highly conserved (at least, in several families of γ-proteobacteria) sequence/structure features, including three hairpins and an unusual Shine-Dalgarno sequence GGGU. Upstream of the SD element, there is an extended (12-nt) AU-rich single-stranded region that serves as a translation enhancer (a presumable target for bS1), and its deletion abolishes translation efficiency (our unpublished data). An important role of the unique conserved 2D/3D structure of the *rplM* 5′UTR in the formation of an autogenous operator for the uL13-repressor has been demonstrated in a more recent study [227].

Interestingly, a recent work [228] has identified a series of *ΔsrmB* suppressor mutations mapped to the 5′UTR of the uL13-uS9 operon, which increases (albeit modestly) expression of both proteins, thereby alleviating the cold-sensitive phenotype of the *ΔsrmB* strain and the assembly defects. These findings suggest that SrmB may be involved in the mechanism that regulates the uL13 production, in addition to its role in forming the 23S binding site for uL13 during the 50S ribosome assembly. However, another recent study [229] has revealed that there is no significant reduction of uL13 in the *ΔsrmB* strain at either 37 °C or 18 °C and, therefore, it is unlikely that a “uL13-limited” assembly pathway underlies cold sensitivity in the absence of SrmB.

### 3.7. bL20, an Autogenous Repressor in E. coli and B. subtilis

The ribosomal protein bL20 belongs to the group of r-proteins (uL3, uL4, uL13, bL20, uL22, and uL24) essential for the first step of the 50S subunit assembly [230]. In *E. coli*, the *rplT* gene encoding bL20 is a part of the *infC* (IF3)-*rpmI* (bL35)-*rplT* (bL20) gene cluster, in which two genes for r-proteins are regulated at the translation level by bL20 [231]. The *infC* gene is not under the bL20-mediated control; rather, it is regulated by IF3. Moreover, in *E. coli*, the promoter for the downstream r-protein genes is located within the *infC* coding region. As a translational repressor, bL20 can bind two sites on the operon mRNA: the first site is represented by a long-range pseudoknot structure, while the second binding site is an irregular hairpin. Both binding sites are important for the bL20 repressor activity in vivo, and together, they bear structural similarity to the bL20 binding site on 23S rRNA, which argues in favor of molecular mimicry [231]. Since the ribosome preferentially binds to the pseudoknot structure, it appears that the autoregulation is based on a competition mechanism [232].

In contrast to *E. coli*, *infC-rpmI-rplT* genes in *B. subtilis* form a real operon that is transcribed from a promoter located upstream of *infC* [233]. The operon is also controlled by bL20, but not at the translation levels in *E. coli*. Two alternative secondary structures may be formed in the mRNA leader upstream of the *infC* translation initiation site, one of which may serve as a transcriptional terminator. The binding of bL20 promotes the formation of the terminator structure that attenuates transcription of the downstream genes. Although the bL20-mediated regulatory mechanism differs from that of *E. coli*, in both cases, the regulation is based on the structural similarity of the bL20 binding regions on the mRNA and 23S rRNA [233]. More recently, it has been shown that removal of the regulatory structure targeted by bL20 in *B. subtilis* results in reduced log-phase growth, improper rRNA maturation, and accumulation of misassembled ribosomal particles at low temperatures, suggesting defects in ribosome biogenesis. This indicates the importance of the autogenous regulation of r-proteins for bacterial fitness [234]. 

The overexpression of bL20 may cause not only autogenous repression. As shown recently, the overexpressed bL20 can partially suppress a cold-sensitive phenotype of the *bipA* null mutant. BipA is a cold-shock inducible GTPase that is pivotal for the 50S ribosomal subunit assembly at low temperatures so that the *bipA*-deleted strain is defective in rRNA processing and 50S biogenesis under these conditions. Ectopic expression of bL20 partially recovers these defects, implying that BipA and bL20 may exert coordinated influence on proper ribosome assembly at low temperatures [235].

### 3.8. Autogenous Regulation of bL25

bL25 is one of the three r-proteins (bL25, uL5, uL18) that interact with 5S rRNA in eubacteria. Unlike the *rplE* (uL5) and *rplR* (uL18) genes that belong to the polycistronic *spc* operon regulated by uS8 at the translation level (see above), the *rplY* gene encoding bL25 is an independent transcription unit (Figure 1). Although bL25 is not essential, *E. coli* cells lacking bL25 reveal a slow-growth phenotype [236,237]. Not all bacteria keep the *rplY* gene in their genomes, e.g., representatives of Fusobacteria, as well as certain lineages of Actinobacteria, Firmicutes, and Tenericutes lack *rplY* [23]. In *rplY*-containing species, proteins of the bL25 family may consist of one (*E. coli* and its closest relatives in γ-proteobacteria) or two domains (all others), with the N-terminal domain being homologous to *E. coli* bL25 and assisting 5S rRNA binding. Why this diversity exists and what is the function of the C-terminal part remains unclear.

In *E. coli* and its relatives possessing a short variant of bL25, the *rplY* expression is regulated in vivo by the mechanism of autogenous repression at the translation level [237]. 5′UTRs of the *rplY* mRNAs from these bacteria bear specific structural and sequence features that are indispensable for autogenous control. The conserved irregular hairpin structure (translational operator) preceding the ribosome binding site and an unusually weak (for highly expressing mRNAs) SD sequence (GAGA), also highly conserved, are crucial for autogenous regulation [237]. Interestingly, these features are only inherent for species with a short bL25. The conversion of a weak GAGA into a classic GGAGG SD element by mutagenesis abolished the autogenous control, which argues in favor of competition between the repressor and a 30S subunit for the *rplY* mRNA. In the presence of a classic SD, the 30S ribosome wins. An analogous situation has been described for the *rpsA* regulation [48].

### 3.9. Dual Activity of bL31 and Its Paralog

Ribosomal protein bL31 is encoded by the *rpmE* gene that forms a monocistronic operon in many bacterial taxa (Figure 1). Though nonessential for bacterial survival under normal growth conditions [17], bL31 plays a crucial role in the formation of the protein–protein intersubunit bridge B1b by interacting with uL5 in a central protuberance of the 50S subunit via its N-terminal domain and with uS13 in a head of the 30S subunit via its C-terminal part [191]. The role of bL31 in the initiation of translation and maintaining the reading frame has also been suggested [238]. Recently, we have shown that bL31, such as many other r-proteins (see above), possesses dual activity in living cells, acting both as an integral ribosome component and a specific autogenous translational repressor [239]. In γ-proteobacteria, the *rpmE* mRNA 5′UTR folds in a secondary structure dedicated to regulation; the structure includes a highly conserved stem–loop element bearing two internal bulges. This conserved irregular hairpin serves as a translational operator targeted by bL31, and the two internal bulges play a critical role so that their elimination by mutations results in a loss of the bL31-mediated translational control. Interestingly, the operator hairpin separates the SD element from the upstream AU-rich translational enhancer (a target for bS1) that is indispensable for the efficient translation of the *rpmE* mRNA but not necessary for autogenous repression. Mutational analysis has revealed that an intrinsically disordered N-terminal segment of bL31 is responsible for its repressor activity [239].

In many bacterial species, a zinc-binding bL31 (also termed bL31A) has a non-zinc-binding paralog, e.g., YkgM in *E. coli* or YtiA in *B. subtilis*, referred to as bL31B [240,241,242,243]. The paralogs have a very modest amino acid identity (less than 40%) but nevertheless occupy the same position on the ribosome. In the log phase under a normal Zn supply, a functionally active paralog is bL31A, while the bL31B synthesis is strongly inhibited by a Zn-dependent transcriptional repressor Zur (zinc uptake regulator). Under Zn deficiency or in a stationary growth phase, the Zur-mediated transcription inhibition is alleviated, leading to an increase in the bL31B cellular concentration. As a result, bL31B displaces bL31A in the ribosome structure [242,243]. Importantly, the *E. coli* paralog bL31B (YkgM) is also able to modulate the *rpmE* expression through a mechanism similar to the autogenous repression by bL31A itself [239,244].

## 4. Non-Specific Activities of Bacterial r-Proteins

### 4.1. Antimicrobial Activity

Intriguingly, sometimes r-proteins exhibit antimicrobial activity, though corresponding information is rather fragmentary, and how r-proteins may act as antimicrobials remain unclear. According to the current hypotheses, r-proteins or their fragments could interfere with the ribosomal assembly of closely related bacteria, or they can induce the production of reactive oxygen species (ROS), with a harmful effect on DNA, RNA, lipids, or proteins of the recipient strain [245]. For instance, the 50S r-proteins bL27 and uL30 of *Lactobacillus salivarius* were shown to possess antimicrobial activity against *Streptococcus pyogenes*, *Streptococcus uberis*, and *Enterococcus faecium* [246]. Antimicrobial peptides from S15, a eukaryotic homolog of bacterial S19, can inhibit bacterial growth and cause bacterial cell destruction, membrane depolarization, and intracellular ROS production [247]. The intriguing antimicrobial actions of r-proteins and their underlying mechanisms deserve further investigation.

### 4.2. Ribosomal Proteins as Chaperons

An important feature of r-proteins is their ability to act as chaperons by assisting in both RNA and protein folding to prevent misfolding into non-functional conformations. Thus, r-proteins uL16, uL18, and bL19 have been shown to act as RNA- and protein-chaperons, and their chaperoning activities towards proteins are comparable to that of the classical Hsp90 chaperone [248]. The moonlighting function as a protein chaperon has been proposed for uS9 [249]. uS9, in concert with UmuC, has been suggested to participate in the error-prone SOS repair process. UmuC plays an important role in the SOS response, and the fact that uS9 can accelerate UmuC renaturation after partial denaturation in vitro has been regarded as an argument for the functional significance of their interaction [249]. However, while the DNA repair mechanisms have been further intensively investigated [250], the role of uS9 in the process has not been discussed anymore.

The role of RNA-chaperoning proteins in bacterial physiology cannot be overestimated. As a rule, RNA can adopt several conformations, with only one being functionally active. Incorrect structures of RNA can lead to inefficient RNA-dependent processes. RNA chaperones resolve such misfolded structures without a requirement for ATP [251]. Certain r-proteins may play a dual role by both stabilizing native rRNA structures and accelerating rRNA refolding during the co-transcriptional assembly of ribosomes. Earlier in vitro experiments demonstrated that uS12 facilitated the splicing of group I introns of phage T4 by preventing the formation of non-catalytic structures without participating in splicing itself, as it could be removed from the reaction mix by proteases before initiation of the splicing by GTP addition [252]. Recent data show that uS12 may fulfill an RNA-chaperoning role in vivo during the 30S co-transcriptional assembly. This conserved late-binding r-protein enhances and accelerates stable binding of the primary r-protein uS4 by acting on the folding path of the nascent rRNA [93].

A prominent example of a ribosomal protein possessing both RNA- and protein-chaperoning activities is bS1 (see Section 2.1). As a protein chaperone, bS1 suppresses a temperature-sensitive missense mutation in a coiled-coil domain of uS2 so that the corresponding *rpsB1^ts^* strain acquires an ability to grow at elevated temperatures in the presence of bS1 expressed from a plasmid [85]. The RNA-chaperone function of bS1 has been widely recognized [253]. An activity of bS1 in remodeling the RNA structure underlies many regulatory pathways at the translation level. It is believed that the intricate coupling of protein and mRNA folding–unfolding dynamics enables translation initiation on structured mRNAs. For bS1 from *Vibrio vulnificus*, RNA-chaperoning activity has been attributed to domains D3 and D4, which provide the mRNA-binding platform, while the D5 domain can significantly increase the chaperoning impact [254]. Further, it has been reported that the ability of riboswitches to regulate translation by inducing metabolite-dependent ON- and OFF-conformations of the ribosome binding site is not based only on the ligand binding but also needs partnership with RNA-binding proteins [255]. In accordance with this, for the adenine-sensing riboswitch from *Vibrio vulnificus*, the synergistic effect of the adenine binding and interaction with bS1 was indispensable for switching to a translational ON-state [256]. bS1 promotes partial mRNA unfolding in other riboswitches as well [45], indicating that the chaperoning activity of bS1 towards the effector domains of translational riboswitches might be an important function of this essential bacterial protein. 

### 4.3. Interactions of r-proteins with DNA

It has been repeatedly observed that certain r-proteins are able to interact with DNA; however, strong evidence of the biological significance of these interactions has not yet been provided. Encoded by the *rpsP* gene, bS16 is an essential bacterial protein that plays an important role in the 30S subunit assembly [257]. The *rpsP* gene belongs to the *trmD* operon, which comprises not only the r-protein genes *rpsP* (bS16) and *rplS* (bL19) but also the genes responsible for the 30S maturation (*rimM*) and tRNA modification (*trmD*) (Figure 1). Up to now, there is no information on the regulation of the operon expression (a rare case among r-protein operons). *E. coli* bS16 was shown to act as a DNase and to interact with the cruciform DNA in solution [258,259]. Cruciform structures are known to influence various aspects of DNA replication and other processes [260]. It remains unclear whether the DNA-nicking activity of bS16 and its ability to bind to the cruciform DNA have any physiological role in the extant bacterial cell or are remnants of previous stages of bacterial evolution when proteins could be adopted by the ribosome from other physiological processes.

Other examples of the DNA-binding r-proteins are represented by bL17, which was found to bind preferentially to curved DNA in *B. subtilis* [261], and uL24, which was isolated as a nucleoid-bound protein again from *B. subtilis* [262]. When overproduced in *E. coli*, *Bsu* bL17 had a strong effect on the nucleoid morphology and segregation. It was suggested that its affinity for curved DNA might be used for some extraribosomal functions [261]. However, no evidence for this has been provided. The condensation of DNA in bacterial nucleoids is a complex and dynamic process in which proteins displaying the properties of histones serve as important contributors. In a search for *B. subtilis* nucleoid-associated proteins, uL24 was identified as an abundant protein in nucleoid-containing fractions [262]. A purified *Bsu* L24 can bind and condense DNA when in vitro. Overexpression of the *rplX* gene encoding uL24 has been shown to disrupt nucleoid segregation and positioning, which hints at the probable extraribosomal function of uL24 as a nucleoid-associated protein. Whether homologous uL24 proteins from other bacterial species can demonstrate analogous nucleoid-binding features remains unexplored. Finally, DNA-binding properties were also ascribed to uL14, which was found to stimulate the ATP-dependent DNA helicase Rep from *E. coli* [263]. The ability of uL14 to increase the rate, as well as the extent of the unwinding reaction catalyzed by Rep, was explained by its interaction with DNA. The binding of uL14 might lower the energy required for the disruption of hydrogen bonds in the double-stranded substrate, making it more favorable for unwinding the duplex region by Rep. Although the data would be of interest, the biological significance of this finding has not been further confirmed.

## 5. Concluding Remarks

The data described in this review show that many bacterial r-proteins have the capacity to function beyond the ribosome (to moonlight), participating in a variety of cellular processes as regulators of translation, transcription, RNA folding and RNA stability, DNA replication, cell wall permeability, and pathogenicity of virulent species. To moonlight, r-proteins interact with other cellular components such as proteins, RNA, membranes, or DNA. Extraribosomal functions of r-proteins have been repeatedly detected over the last decades in all kingdoms of life [22,264,265,266]. As widely accepted, to conclude that a certain r-protein possesses moonlighting activities, three criteria should be considered as follows: (i) the r-protein interacts specifically with the non-ribosomal component of the cell; (ii) such interaction has a physiological effect; and (iii) the interaction occurs outside the ribosome [265,266]. If we are to follow these criteria, not all r-proteins described in this review are, strictly speaking, moonlighters. For instance, bS21 regulates its own synthesis in Flavobacteria or discriminates certain mRNAs in *F. tularensis* as an integral part of the ribosome, not outside (see Section 2.10).

However, such an unforeseen regulatory function of the r-protein deserves to be described, given that until now, our knowledge of functional specificities of ribosomal components is incomplete, and much is still unknown about the mechanisms by which r-proteins may act within, not to mention beyond the ribosome. To find and characterize novel extraribosomal activities of r-proteins, it is highly desirable to have a notion about their specific functions in translation. It is noteworthy, however, that r-proteins with moonlighting activities appear, as a rule, more stable, even during extended stationary-phase growth, while other r-proteins are subject to rapid degradation as soon as rRNA is degraded [267]. Remarkably, some r-protein moonlighters can perform several different functions beyond the ribosome, being really polyfunctional cellular components (e.g., bS1, uS4, uL4). The list of moonlighting r-proteins has expanded significantly during the last decade but remains, no doubt, far from completeness, even for well-studied model bacteria like *E. coli* or *B. subtilis*. There is every reason to believe that many potential moonlighters with a previously unrecognized role outside the ribosome will be discovered in future studies of both model and yet poorly explored bacterial species.

## Data Availability

Not applicable.
